# Supplementary material for: Splice-Junction-Based Mapping of Alternative Isoforms in the Human Proteome
Source: Cell Rep. Author manuscript; Available in PMC 2020 Jan 15. (PMC6961840; doi:10.1016/j.celrep.2019.11.026)
Supplement: 2 [file NIHMS1546469-supplement-2.zip › DF1/PXD006675/AtrialSeptum/AtrialSeptum_6_UQCRH_MLTESGDPEEDPLTTVR.pdf]

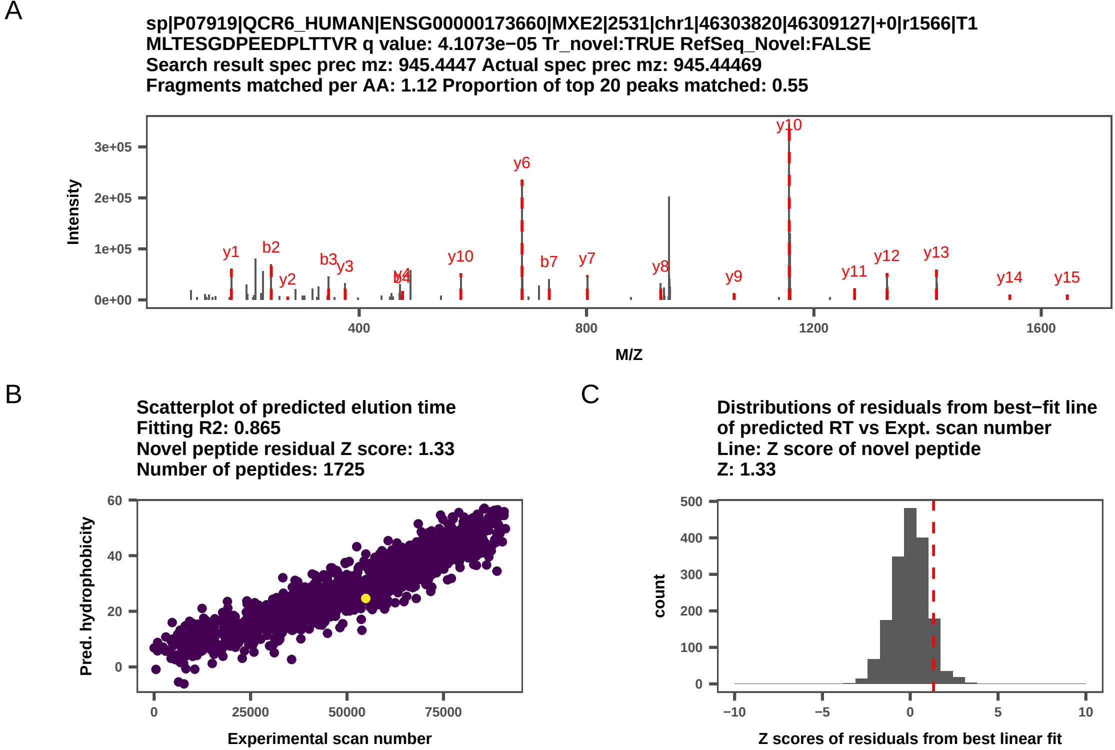
A
sp|P07919|QCR6_HUMAN|ENSG00000173660|MXE2|2531|chr1|46303820|46309127|+0|r1566|T1
MLTESGDPEEDPLTTVR q value: 4.1073e−05 Tr_novel:TRUE RefSeq_Novel:FALSE
Search result spec prec mz: 945.4447 Actual spec prec mz: 945.44469
Fragments matched per AA: 1.12 Proportion of top 20 peaks matched: 0.55
Intensity
3e+05
2e+05
1e+05
0e+00
y1
b2
y2
b3
y3
y4
b4
y10
y6
b7
y7
y8
y9
y10
y11
y12
y13
y14
y15
400
800
1200
1600
M/Z
B
Scatterplot of predicted elution time
Fitting R2: 0.865
Novel peptide residual Z score: 1.33
Number of peptides: 1725
Pred. hydrophobicity
60
40
20
0
0
25000
50000
75000
Experimental scan number
C
Distributions of residuals from best−fit line
of predicted RT vs Expt. scan number
Line: Z score of novel peptide
Z: 1.33
count
500
400
300
200
100
0
−10
−5
0
5
10
Z scores of residuals from best linear fit
